# Prevalence, Risk Factors, and Impact on Quality of Life Due to Urinary Incontinence Among Palestinian Women: A Cross-Sectional Study

**DOI:** 10.7759/cureus.57813

**Published:** 2024-04-08

**Authors:** Faris Abushamma, Assil Mansour, Rula Nassar, Huda Badran, Rola Abu Alwafa, Mahfouz Ktaifan, Sa’ed H Zyoud

**Affiliations:** 1 Medicine, An-Najah National University, Nablus, PSE; 2 Urology, An-Najah National University Hospital, Nablus, PSE; 3 General Practice, An-Najah National University, Nablus, PSE; 4 Urology, An-Najah National University, Nablus, PSE; 5 Internal Medicine, An-Najah National University, Nablus, PSE

**Keywords:** quid, iciq-ui, iiq-7, quality of life, urinary incontinence

## Abstract

Introduction: Urinary incontinence (UI) is a common condition that affects females with variable incidence. Factors like age, obesity, weak pelvic floor, and pregnancy contribute to UI pathogenesis. Our study aimed to determine the prevalence of UI and identify associated risk factors.

Methods: A cross-sectional study recruited females aged 18-65 attending primary health care (PHC) centers. The collected data included demographic information and questionnaire scores for urinary incontinence diagnosis (QUID), International Consultation on Incontinence Questionnaire-Urinary Incontinence (ICIQ-UI), and Incontinence Impact Questionnaire-Short Form (IIQ-7) scores.

Results: Three hundred and eleven females met our inclusion criteria, with 162 (52.1%) participants aged ≥ 42 years. Approximately 41.5% were college/university graduates, and 23.2% had an education level less than high school. Moreover, 108 (34.7%) participants were smokers, 223 (71.7%) drank coffee, and approximately 212 (68.2%) drank tea. Only 125 (40.2%) participants engaged in exercise at variable frequencies, and most of them exercised once per week. Approximately 27.3% of the participants had chronic medical illness with hypertension or diabetes mellitus (40 (12.9%) or 25 (8%), respectively). Stress urinary incontinence (SUI) was found among 152 (48.9%) participants, while urgency urinary incontinence (UUI) was found among 114 (36.7%) participants. Age ≥ 42, marital status, low educational level, unemployment, lack of physical activity, and chronic medical illnesses were significantly correlated with both SUI and UUI. There was a strong correlation between UI and the severity of symptoms and between UI and quality of life (QoL).

Conclusion: The prevalence of UI is relatively high among Palestinian women. Many factors contributing to UI included age, marital status, the presence of other chronic medical diseases, and a lack of physical activity. Early detection and diagnosis are necessary to provide effective treatment and improve UI symptoms and QoL.

## Introduction

The International Continence Society has defined urinary incontinence (UI) as the complaint of involuntary loss of urine [1]. Several types of urinary incontinence have also been described, and at most, our study focused on stress urinary incontinence (SUI) and urgency urinary incontinence (UUI).

SUI is a condition characterized by the involuntary loss of urine during physical exertion, such as sneezing, coughing, or engaging in sporting activities, and is not related to psychological stress, whereas UUI has been described as the involuntary loss of urine associated with urgency [1]. The factors contributing to UI include advanced age, chronic cough, connective tissue disorders, constipation, heavy lifting, menopause, obesity, pelvic floor trauma, pregnancy, and smoking. Previous pelvic surgeries can also lead to neuromuscular damage [2,3].

Studies from different countries highlight the prevalence and risk factors for UI among women. In Jordan, more than half of the participants experienced SUI, emphasizing the need for early diagnosis and treatment [3]. Chinese studies revealed diverse prevalence rates associated with UI and age, BMI, malnutrition status, education, and medical conditions [4,5]. In France, nearly one in four women visiting family physicians exhibited UI symptoms, with age, BMI, and parity influencing severity [6]. A Tunisian study showed that 45.3% of women experienced urinary or anal incontinence, citing risk factors such as postpartum urinary incontinence, menopausal status, arterial hypertension (HTN), nurse occupation, and constipation [7]. In Saudi Arabia, multiple studies have presented diverse findings on the prevalence of UI. The overall prevalence of UI ranged from 41.1% to 56.6% across different studies [8-12]. Specifically, SUI rates were noted at 15.4%, 20%, and 3.3% in individual studies [9,12,13]. Furthermore, various studies have reported that UUI has been acknowledged by roughly less than a third of affected women. [8,9,11].

Moreover, UI is a common and distressing condition that significantly affects the QoL of young females. Studies have shown that SUI can cause social and sexual limitations [14]. Pelvic exercises that strengthen the pelvic floor muscles are also known to prevent SUI [15]. Additionally, SUI and UUI can be managed through both medical and surgical interventions [16,17].

Our group has published several papers about lower urinary tract symptoms (LUTS) and UI among different cohorts of patients in Palestine [18-22]. This was a national study investigating the prevalence of UI and the factors that influence it.

## Materials and methods

Study design and setting

This was a cross-sectional study in which females attending primary health care (PHC) centers in the West Bank were recruited.‎ Convenience sampling was employed in this study, and the data were collected between April 2022 and December 2022.

Study population, sampling procedure, and sample size calculation

The research participants were women who were attending PHC clinics. The sample size of 300 was determined by using an online Raosoft sample size calculator. This calculation resulted in a convenient sample with a confidence level of 95% and a margin of error of 5%.

Inclusion and exclusion criteria

The inclusion criteria were females aged between 18 and 65 who attended PHC centers. The exclusion criteria were patients with urinary tract anomalies, documented or symptomatic urinary tract infections, medications that affect urinary bladder function, a history of chronic neurological disease, or a history of or current major psychiatric illness.

Data collection instruments

The data collection instrument utilized was a questionnaire in the Arabic language organized into four sections as outlined below:

Demographics and Clinical Data

Age, employment status, marital status, and education level were included. Smoking status, caffeine intake, medication use, history of previous pelvic surgeries, presence of chronic medical illness, physical activity, number of both vaginal and cesarean deliveries, number of delivered babies above 4 kg, and age at first delivery were also collected.

The Questionnaire for Urinary Incontinence Diagnosis (QUID)

The Questionnaire is a 6-item survey designed to assess UI symptoms [23]. It was developed and validated specifically to differentiate between stress and urge UI. It consists of 6 questions. The stress score is calculated by summing the responses to items 1, 2, and 3, while the urge score is determined by summing the responses to items 4, 5, and 6. The intensity of each symptom is classified on a scale from 0 to 5.

International Consultation on Incontinence Questionnaire-Urinary Incontinence (ICIQ-UI)

The ICIQ-UI short form [24] is a questionnaire for evaluating the severity, frequency, and impact of UI on QoL in both sexes in clinical and research practices worldwide. The total score represents the sum of all symptoms, with an overall score of 21 and greater values indicating increased symptom severity.

Incontinence Impact Questionnaire, Short Form (IIQ-7)

The IIQ-7 [25] is a tool specifically designed to evaluate the influence of UI on an individual's QoL. The assessment focused on four key areas: physical activity, travel, social relations, and emotional well-being. Symptom severity was graded on a scale from zero to three, with zero indicating the least severe and three indicating the most severe. The maximum IIQ-7 score achievable is 21, and a higher score corresponds to a more significant impact on QoL. The Cronbach alpha coefficient was employed to validate this tool, demonstrating an impressive internal consistency of 0.98 [26].

Ethics approval and consent to participate

The study protocol, encompassing access to and utilization of clinical information, received approval from the Institutional Review Boards (IRBs) of An-Najah National University; the IRB approval number is Med. August, 2022/22. The research adhered to ethical standards established by the Human Experimentation Responsible Committee (at the institutional and national levels) and the Helsinki Declaration. All participants provided verbal informed consent for their involvement in the study, and they were informed about the study's objectives and their potential contribution to UI research before giving consent.

Statistical analysis

The information was analyzed utilizing version 21 of the Social Sciences Statistical Package (IBM-SPSS). Continuous variables are presented as the means and standard deviations, while categorical variables are expressed as frequencies and percentages. Correlations were assessed using the Pearson test. To evaluate the significance of differences between categorical variables, the chi-square test or Fisher’s exact test was employed as appropriate. Differences in means between categories were tested using the Kruskal‒Wallis test followed by Bonferroni-Dunn post hoc analysis or the Mann‒Whitney test. A p-value less than 0.05 was considered to indicate statistical significance.

## Results

Demographics and Clinical Data

Three hundred and eleven females met our inclusion criteria; 162 (52.1%) of the participants were ≥ 42 years old. Two hundred thirty-five (75.6%) of the females were married, while only 35 (11.3%) were single. Regarding educational level, 129 (41.5%) were college/university graduates, and 72 (23.2%) had an education level lower than high school. Most of the participants were unemployed 108 (34.7%), followed by employed full-time (40 hours or more weekly) (102, 32.8%). Moreover, of the 108 (34.7%) smokers, 48 (15.4%) and 79 (25.4%) smoked cigarettes and shisha, respectively. Coffee drinking was the predominant drinking mode, with approximately 223 (71.7%) drinking coffee. Tea consumption accounted for approximately 212 (68.2%) of the sample. Only 125 (40.2%) participants exercised at variable frequencies; most exercised once weekly. Approximately 27.3% of the participants had chronic medical illness with HTN or diabetes mellitus (DM) (40 (12.9%) or 25 (8%), respectively). In addition, 227 (73%) patients were off medications, and 102 (32.8%) had prior pelvic surgeries. The median age at first delivery was 21 years (19-24). The median overall number of deliveries was 3 (2-4), the median number of vaginal deliveries was 3 (2-4), and the median number of cesarean deliveries was 0 (0-1). Moreover, 64 (20.6%) delivered babies were 4kg or more (Table 1).

**Table 1:** Demographics and clinical data of the study participants Data is represented as either Frequency (%) or median [Q1-Q3]

Demographics and clinical data		Frequency (%) or median [Q1-Q3] N = 311
Age		42 (33 – 52)
Age	<42	149 (47.9)
	≥ 42	162 (52.1)
Marital Status	Single	35 (11.3)
	Married	235 (75.6)
	Separated	14 (4.5)
	Widow	27 (8.7)
Educational Level	Less than high school	72 (23.2)
	High school graduate	110 (35.4)
	College/University graduate	129 (41.5)
Occupational Status	Unemployed	108 (34.7)
	Student	22 (7.1)
	Self-employed	23 (7.4)
	Full-time employed (40 hours or more weekly)	102 (32.8)
	Partial-time employed (less than 40 hours a week)	46 (14.8)
	Retired	10 (3.2)
Smoker	Yes	108 (34.7)
	No	203 (65.3)
Cigarettes	Yes	48 (15.4)
	No	263 (84.6)
Shisha	Yes	79 (25.4)
	No	232 (74.6)
Coffee	Yes	223 (71.7)
	No	88 (28.3)
Coffee cups per day		2 (1 -3)
Tea	Yes	212 (68.2)
	No	99 (31.8)
Tea cups per day		2 (1 -3)
Exercise	Yes	125 (40.2)
	No	186 (59.8)
Exercise Frequency	Once a day	13 (4.2)
	Every other day	46 (14.8)
	Once a week	56 (18)
	Once a month	10 (3.2)
Chronic Medical Condition	Yes	85 (27.3)
	No	226 (72.7)
Chronic Medical Condition Type	Hypertension	40 (12.9)
	Diabetes	25 (8)
	Cardiovascular diseases	3 (1)
	Malignancy	2 (0.6)
	Respiratory diseases	1 (0.3)
	Immunological condition	2 (0.6)
	Other	12 (3.9)
Medications	Yes	84 (27)
	No	227 (73)
Prior Pelvic Surgery	Yes	102 (32.8)
	No	209 (67.2)
Newborn Weighted 4 kg or more	Yes	64 (20.6)
	No	110 (35.4)
Number of vaginal deliveries		3 (2-4)
Number of cesarean deliveries		0 (0-1)
Number of miscarriage abortions		0 (0-0)
Age at which you gave first birth		21 (19-24)
Number of deliveries you have had		3 (2-4)

The Questionnaire for Urinary Incontinence Diagnosis (QUID)

SUI was found among 152(48.9%) participants, while UUI was found among 114 (36.7%) participants. Age ≥ 42 was significantly correlated with both SUI and UUI (102 (67.1%) and 79 (69.3%, respectively; p<0.001)). Marital status was significantly correlated with both variables; 118 (77.6%) of the married females had SUI, and 88 (77.2%) had UUI (p<0.001). SUI was predominant among females with a low education level (54 (35.5%) for both low and high school graduates). Moreover, a level of education less than high school was positively correlated with UUI 44 (38.6%) (p<0.001). Unemployed females were more likely to have SUI and UUI (62 (40.8%) (p=0.01) and 50 (43.9%) (p=0.006), respectively). Coffee drinking was only associated with SUI 119 (78.3%) (p=0.012). Not exercising was associated with SUI (108 (71.1) (p<0.001)) and UUI (78 (68.4%); p=0.018). Chronic medical diseases were positively correlated with SUI and UUI; 31(59.6%) hypertensive females and 17 (32.7%) diabetic females had SUI (p<0.001), and 27(57.4%) hypertensive and 15(31.9%) diabetic patients had UUI (p=0.005). Smoking status, tea consumption, and history of pelvic surgery were not correlated with either SUI or UUI (Table 2).

**Table 2:** Associations between stress and urgency incontinence scale scores and demographic data Data is represented as Frequency (%) ^b ^Statistically significant values were calculated using the Pearson chi-square test. ^c ^Statistically significant values were calculated using Fisher's exact test. CVD: Cardiovascular diseases

Variables		Patient with Stress incontinence frequency (%) n=152 (48.9)	Patient with Urgency incontinence frequency (%) n=114 (36.7)
Age	<42	50(32.9)	35(30.7)
	≥42	102(67.1)	79(69.3)
	*p-*value	<0.001^b^	<0.001^b^
Marital status	Single	7(4.6)	5(4.4)
	Married	118(77.6)	88(77.2)
	Separated	7(4.6)	4(3.5)
	Widow	20(13.2)	17(14.9)
	*p-*value	<0.001^b^	0.001^c^
Educational Level	Less than high school	54(35.5)	44(38.6)
	High school graduate	54(35.5)	41(36)
	College/University graduate	44(28.9)	29(25.4)
	*p-*value	<0.001^b^	<0.001^b^
Occupational status	Unemployed	62(40.8)	50(43.9)
	Students	8(5.3)	3(2.6)
	Self- employed	11(7.2)	7(6.1)
	Full-time employed (40 hours or more weekly)	51(33.6)	38(33.3)
	Partial time	13(8.6)	10(8.8)
	Retired	7(4.6)	6(5.3)
	*p-*value	0.01^ b^	0.006^ c^
Smoking	Yes	58(38.2)	43(37.7)
	No	94(61.8)	71(62.3)
	*p-*value	0.214^ b^	0.399^b^
Coffee drinking	Yes	119(78.3)	86(75.4)
	No	33(21.7)	28(24.6)
	*p-*value	0.012^b^	0.266^b^
Tea drinking	Yes	111(73)	84(73.7)
	No	41(27)	30(26.3)
	*p-*value	0.072^ b^	0.112^b^
Exercise	Yes	44(28.9)	36(31.6)
	No	108(71.1)	78(68.4)
	*p-*value	<0.001^b^	0.018^ b^
Chronic medical condition	Hypertension	31(59.6)	27(57.4)
	Diabetes	17(32.7)	15(31.9)
	CVD	3(5.8)	3(6.4)
	Malignancy	0(0)	0(0)
	Respiratory diseases	0(0)	0(0)
	Immunological conditions	0(0)	0(0)
	Others	1(1.9)	2(4.3)
	*p-*value	<0.001^c^	0.005^ c^
Prior Pelvic surgery	Yes	55(36.2)	41(36)
	No	97(63.8)	73(64)
	*p-*value	0.214^ b^	0.365^ b^

International Consultation on Incontinence Questionnaire-Urinary Incontinence (ICIQ-UI)

Age was significantly correlated with the ICIQ-UI score, with a median of 9 (7-12) for patients aged≥ 42 years and a median of 1 (1-6.5) for patients aged<42 years (p<0.001). The ICIQ-UI score was significantly greater among widow females, with a median of 13 (9-14) than among single females, with a median of 4 (1-10.25) (p<0.001). Individuals with less than a high school education had the highest ICIQ-UI score, with a mean rank of 194.33 (p<0.001). Moreover, retired females had the highest score, with a median of 10 (7-18). Tea consumption was significantly correlated with an ICIQ-UI score of 9 (5.75-11) (p=0.011). Not exercising was associated with ICIQ-UI, with a mean rank of 166.27. Furthermore, prior pelvic surgery was associated with a median ICIQ-UI of 8.5 (4.75-12.25) (p=0.008). In regards to chronic medical illness, patients with cardiovascular diseases (CVD), HTN, and DM had higher scores of ICIQ-UI with a mean rank of (72.83, 47.81, 46.60) respectively (p=0.002). In contrast, smoking and drinking coffee were not associated with ICIQ-UI (Table 3).

**Table 3:** Associations between ICIQ-UI scores and demographic and clinical data Data is represented as Median (Q1-Q3) and Mean rank: ^b ^Statistically significant values were calculated using the Mann–Whitney U test. ^c ^Statistically significant values were calculated using the Kruskal‒Wallis test. CVD: Cardiovascular diseases

	Variables	Mean rank	Median (Q1-Q3)	*P *value
Age	<42	117.87	1(1-6.5)	<0.001^b^
	≥42	191.07	9(7-12)	
Marital status	Single	94.80	4(1-10.25)	<0.001^c^
	Married	160.74	8(5.25-10)	
	Separated	151.25	5(1.25-8)	
	Widow	196.57	13(9-14)	
Educational Level	Less than high school	194.33	9(7-11)	<0.001^c^
	High school graduate	160.70	9(4.25-13.75)	
	College/University graduate	130.60	6(1-8.75)	
Occupational status	Unemployed	166.88	9(7-12.25)	0.014^ c^
	Students	111.73	0(0-0)	
	Self- employed	153.72	6(1-12)	
	Full-time employed (40 hours or more weekly)	160.60	8.5(1.75-11)	
	Partial time	131.40	4(1-8.5)	
	Retired	207.40	10(7-18)	
Smoking	Yes	167.51	8(6-11)	0.095^b^
	No	149.87	8.5(1-11)	
Coffee drinking	Yes	160.20	8(5-10)	0.184^b^
	No	145.37	9(3.5-13.25)	
Tea drinking	Yes	164.68	9(5.75-11)	0.011^b^
	No	137.40	7(1-11)	
Exercise	Yes	140.72	8(1.75-14)	0.013^b^
	No	166.27	8(5-11)	
Chronic medical condition	Hypertension	47.81	9(7.25-11)	0.002^C^
	Diabetes	46.60	9(5-12.5)	
	CVD	72.83	17(13.5-17.5)	
	Malignancy	10.00	1(1-1)	
	Respiratory diseases	10.00	0(0-0)	
	Immunological conditions	16.50	3(1-5)	
	Others	24.67	4.5(1-7.75)	
Prior Pelvic surgery	Yes	174.97	8.5(4.75-12.25)	0.008^ b^
	No	146.74	8(2-11)	

Incontinence Impact Questionnaire (IIQ-7)

The greater the IIQ-7 score was, the greater the impact of urinary incontinence on QoL. Age ≥ 42 years was significantly correlated with the IIQ-7 score, with a median of 57.08 (23.78-66.6) (p<0.001). Widow females, those with less than a high school education, and retired females had higher IIQ-7 scores, with mean ranks of 192.56, 209.39, and 212.00, respectively (p<0.001). The IIQ-7 score was greater among females who did not exercise, with a median of 52.32 (16.65-66.6) (p<0.001). Moreover, there was a positive correlation between the IIQ-7 score and chronic medical diseases; CVD, HTN, and DM participants had higher scores, with a mean rank of 68.33 for CVD, 49.70 for HTN, and 45.60 for DM (p=0.001). Smoking status, drinking of coffee, drinking of tea, and prior pelvic surgery did not reach statistical significance (Table 4).

**Table 4:** Associations between the IIQ-7 score and demographic and clinical data Data is represented as Median (Q1-Q3) and Mean rank: ^b ^Statistically significant values were calculated using the Mann–Whitney U test ^c ^Statistically significant values were calculated using the Kruskal‒Wallis test

	Variables	Mean rank	Median (Q1-Q3)	*P* value
Age	<42	126.79	0(0-14.27)	<0.001^b^
	≥42	182.87	57.08(23.78-66.6)	
Marital status	Single	103.36	19.02(0-66.60)	<0.001^c^
	Married	159.29	47.57(1.18-66.6)	
	Separated	161.96	7.13(0.0-53.51)	
	Widow	192.56	66.6(47.57-80.87)	
Educational Level	Less than high school	209.39	66.6(47.57-71.35)	<0.001^c^
	High school graduate	158.56	52.32(0.0-56.41)	
	College/University graduate	124.02	11.89(0.00-28.54)	
Occupational status	Unemployed	175.97	57.08(33.3-66.6)	<0.001^c^
	Students	111.45	0(0-0)	
	Self- employed	157.65	14.27(0-61.84)	
	Full time employed (40 hours or more weekly)	156.54	33.3(0-66.6)	
	Partial time	116.22	0(0-33.3)	
	Retired	212.00	71.35(23.7-95.14)	
Smoking	Yes	166.45	42.81(4.75-66.6)	0.123^b^
	No	150.44	47.57(0-66.6)	
Coffee drinking	Yes	161.20	47.57(0-66.6)	0.093^b^
	No	142.82	54.7(0-67.78)	
Tea drinking	Yes	160.28	54.7(0-66.6)	0.204^b^
	No	146.83	33.3(0-66.6)	
Exercise	Yes	13.66	14.27(0-66.6)	<0.001^b^
	No	173.03	52.32(16.65-66.6)	
Chronic medical condition	Hypertension	49.70	57.08(28.54-66.6)	0.001^c^
	Diabetes	45.60	57.08(7.13-66.6)	
	CVD	68.33	95.14(66.6-97.52)	
	Malignancy	11.50	0(0-0)	
	Respiratory diseases	26.50	0(0-0)	
	Immunological conditions	11.50	0(0-0)	
	Others	20.79	2.37(0-14.27)	
Prior Pelvic surgery	Yes	164.37	33.3(0-71.35)	0.236^b^
	No	151.91	52.32(4.75-66.6)	

Correlations Between the Severity of Urinary Incontinence (ICIQ-UI), Stress UI, Urgency UI, and IIQ-7 Scores

There was a strong correlation between SUI and the severity of incontinence measured by the ICIQ-UI (r=0.855) (p<0.001). Furthermore, there was a statistically significant correlation between the severity of IIQ-7, UUI, and the severity of UI (r=0.796 and r=0.812; p<0.001, respectively) (Table 5).

**Table 5:** Correlations between the severity of urinary incontinence and stress UI, urgency UI and IIQ-7 scores **Correlation is significant at the 0.01 level (2-tailed). Correlation measured by the Pearson correlation coefficient (r) UI: Urinary incontinence; ICIQ: International Consultation on Incontinence Questionnaire; IIQ: Incontinence Impact Questionnaire

N=311	P value	Pearson Correlation
ICIQ and Stress scale	<0.001	0.855**
ICIQ and Urgency scale	<0.001	0.812**
ICIQ and IIQ-7 scale	<0.001	0.796**

## Discussion

The present study investigated the demographics and clinical characteristics of female participants, their correlation with different types of UI, and the impact of UI on their QoL. The findings provide valuable insights into this cohort's prevalence and risk factors associated with UI.

Our study showed that SUI was prevalent among 152 (48.9%) participants, while UUI was found among 114 (36.7%) participants. These findings are consistent with those of previous studies in which the incidence of SUI was 55.1% in the Jordanian cohort [3], while a Tunisian study showed 40.3% UUI, 24.6% SUI, and 19.9% mixed UI [7]. Other studies have shown that variations in UI prevalence and risk factors would vary depending on different cultural or geographical contexts [27]. 

The majority of participants were aged 42 years or older, with more than half of the sample falling into this age group. This finding aligns with age-related trends in UI incidence [28-30]. Marital status was significantly correlated with SUI and UUI incidence. Married females exhibit a greater likelihood of UI, possibly due to factors such as pregnancy and hormonal changes [31-33]. In an American study, multiparity was identified as a significant risk factor for SUI compared to uniparity or nulliparity. Complicated labor was significantly more strongly associated with UI than uncomplicated labor [32].

Smoking status, which was not significantly different in our study, was strongly associated with SUI according to previous studies, with a reported relative risk ranging from 1.8 to 2.92 [34-36]. Physical inactivity correlates with an increased incidence of both SUI and UUI, underscoring the role of exercise in pelvic floor strength and UI risk reduction [37].

Educational level correlates with UI, as lower educational levels are associated with higher incidences of SUI and UUI, emphasizing the importance of targeted educational interventions to improve awareness and promote early intervention for UI [38]. Employment status is linked to SUI and UUI, with a higher prevalence among unemployed females, aligning with previous research on unemployment as a UI risk factor [39,40]. Chronic medical conditions, HTN, and DM were positively correlated with SUI and UUI, consistent with previous findings identifying these conditions as UI risk factors [41,42].

Tea consumption was significantly correlated with higher ICIQ-UI scores, suggesting an association with the impact of UI on QoL. The caffeine content in tea, approximately one-third of that in coffee, prompted the exploration of other components potentially associated with lower urinary tract dysfunction [34,43]. Prior pelvic surgery is associated with higher ICIQ-UI scores, indicating a greater impact on QoL, possibly through structural or functional changes in the pelvic region [44].

Factors such as not engaging in exercise, HTN, and DM were associated with higher IIQ-7 scores, indicating a greater impact of UI on daily activities. Managing these modifiable risk factors may positively influence the overall impact of UI on daily life [18,45,46].

Strengths and limitations of our study

This study marks the first exploration of the prevalence and severity of urinary incontinence in Palestinian females. However, the cross-sectional design limits our ability to establish a cause-effect relationship, introducing potential biases. Thus, longitudinal studies could establish causality between identified risk factors and UI for future research direction. Additionally, we do not have BMI data for all patients, as many women in our countries are unwilling to declare their weight for research purposes.

## Conclusions

Our study revealed a notable prevalence of UI, particularly SUI and UUI, among Palestinian females in the West Bank. Age, marital status, education, employment, physical activity, and chronic medical conditions were identified as significant factors correlated with both SUI and UUI. The strong association between these factors and the severity of UI symptoms underscores the complexity of this condition. These findings advocate for targeted interventions addressing modifiable risk factors to enhance prevention and management strategies, ultimately improving the overall QoL for affected Palestinian women.
